# Innovative Approaches to Menstruation and Fertility Tracking Using Wearable Reproductive Health Technology: Systematic Review

**DOI:** 10.2196/45139

**Published:** 2024-02-15

**Authors:** Lynnette Lyzwinski, Mohamed Elgendi, Carlo Menon

**Affiliations:** 1 Menrva Research Group, School of Mechatronics Systems Engineering and Engineering Science Simon Fraser University Vancouver, BC Canada; 2 Biomedical and Mobile Health Technology Laboratory Department of Health Sciences and Technology ETH Zurich Zurich Switzerland

**Keywords:** fertility cycle, fertility monitoring, ovulation, menstruation, wearable devices, mHealth, reproductive health, wearable, fertility, menstrual, women’s health, ovulate, sexual health, scoping, review method

## Abstract

**Background:**

Emerging digital health technology has moved into the reproductive health market for female individuals. In the past, mobile health apps have been used to monitor the menstrual cycle using manual entry. New technological trends involve the use of wearable devices to track fertility by assessing physiological changes such as temperature, heart rate, and respiratory rate.

**Objective:**

The primary aims of this study are to review the types of wearables that have been developed and evaluated for menstrual cycle tracking and to examine whether they may detect changes in the menstrual cycle in female individuals. Another aim is to review whether these devices are effective for tracking various stages in the menstrual cycle including ovulation and menstruation. Finally, the secondary aim is to assess whether the studies have validated their findings by reporting accuracy and sensitivity.

**Methods:**

A review of PubMed or MEDLINE was undertaken to evaluate wearable devices for their effectiveness in predicting fertility and differentiating between the different stages of the menstrual cycle.

**Results:**

Fertility cycle–tracking wearables include devices that can be worn on the wrists, on the fingers, intravaginally, and inside the ear. Wearable devices hold promise for predicting different stages of the menstrual cycle including the fertile window and may be used by female individuals as part of their reproductive health. Most devices had high accuracy for detecting fertility and were able to differentiate between the luteal phase (early and late), fertile window, and menstruation by assessing changes in heart rate, heart rate variability, temperature, and respiratory rate.

**Conclusions:**

More research is needed to evaluate consumer perspectives on reproductive technology for monitoring fertility, and ethical issues around the privacy of digital data need to be addressed. Additionally, there is also a need for more studies to validate and confirm this research, given its scarcity, especially in relation to changes in respiratory rate as a proxy for reproductive cycle staging.

## Introduction

### Overview

Digital technology has been developed over the years to allow female individuals to monitor and take control of their reproductive health. Technological developments that may assist female individuals in monitoring their fertility cycle hold great potential for preventing unplanned pregnancy and in helping female individuals achieve planned pregnancy.

To date, most research has focused on commercially available smartphone apps for fertility monitoring [1]. However, a previous review found that not all commercially available apps are evidence based [1]. Furthermore, most focus on tracking menstruation by the user manually entering data into the app and self-checking temperature [1,2]. There may be disadvantages to this method, including reporting errors, the need to recall dates concerning menstruation, and cycle irregularity, which may affect the accuracy of predicting the next cycle dates [3,4]. One study of 330 female individuals found that periodic tracking apps had made errors in predicting periods, with 57% (n=189) of female individuals having experienced an earlier period than estimated, while 72% (n=238) had a delayed period relative to what was anticipated [5]. Some reviews have also critiqued menstrual cycle–tracking apps for lacking evidence of their efficacy at times [1].

Recently, there have been technological advancements in this field that involve the detection of fertility and menstruation based on heart rate variability (HRV) as well as temperature changes [6] using wearable devices [7-13]. For example, the Oura Ring tracks menstruation through changes in the sleep cycle, heart rate (HR), and temperature [7,9,14]. The Ava bracelet is another novel wearable piece of smart jewelry that checks HRV to predict fertility [10]. These wearable devices predict menstrual cycle stages based on physiological changes, including differentiation between the luteal phase, ovulation, and menstruation.

The female reproductive cycle is typically 28-30 days, although there is variability in some female individuals who may have shorter or longer cycles [15]. The shedding of the endometrial lining in the uterus results in menstruation, formally classified as day 1 of the cycle [16], which takes place over a period of 4-6 days on average in most female individuals, although some have longer or shorter periods [15]. The follicular phase begins at the onset of menses as follicles begin to grow, with a rise in estrogen midphase [15] and with subsequent ovulation and release of an egg at the next stage at approximately 12-14 days before menstruation [17], although some female individuals may ovulate earlier at day 7 [18]. The fertile window is when a female individual is most fertile within 5 days before ovulation, accounting for sperm viability, and during ovulation when the egg is released and viable over 24 hours [16,19]. Luteinizing hormones (LH) surge when a female individual is ovulating and basal body temperature increases by 0.3 °C [20,21]. The fourth stage of the cycle, the luteal phase, lasts approximately 14 days, which is accompanied by rising progesterone levels and the midphase rise of estrogen, with thickening of the uterus for implantation [15,20]. The onset of the new cycle with menstruation occurs once again de novo, with drops in progesterone and estrogen if the egg is not fertilized along with a drop in body temperature back to regular levels [15,22].

Gaining insight into fertility in real time may be a very instructive form of fertility care, as well as a useful tool for reproductive health in female individuals. By monitoring their cycle through body temperature changes, female individuals may either best prevent or plan a pregnancy when they know when they are ovulating. It may also help female individuals with managing their menses by being aware of when they may expect their period as well as with general cycle and stage monitoring. However, little is known about whether these devices are accurate predictors of fertility cycle staging from ovulation to menses. It is also useful to know whether certain physiological parameters or a specific combination best predict fertility (eg, temperature alone vs temperature and HR). Additionally, it is essential to compare the types of wearables that are available, including their design and placement on the body.

Thus, there is a need to undertake a comprehensive review to evaluate what types of emerging wearable technologies for fertility cycle monitoring have been developed. It is of research interest to better understand where these devices may be worn and which physiological parameters are used to track fertility at different stages. In other words, what has been developed and evaluated for efficacy and accuracy, and what can be improved in future technological developments?

### Aims

The primary aims of this study are to review the types of wearables that have been developed and evaluated for menstrual cycle tracking and to examine whether they may detect changes in the menstrual cycle in female individuals. Another aim is to review whether these devices are effective for tracking various stages in the menstrual cycle including ovulation and menstruation. Finally, the secondary aim is to assess whether the studies have validated their findings by reporting accuracy, sensitivity, or positive predictive value.

## Methods

### Study Guidelines

This review was conducted according to the PRISMA (Preferred Reporting Items for Systematic Reviews and Meta-Analyses) statement [23]. A prior review protocol was drafted using the PRISMA Protocols for internal use among the research team but it was not externally published or registered prospectively.

### Search Strategy and Study Eligibility

PubMed or MEDLINE was searched for papers published between July 1, 2012, and July 1, 2022, for all English-language papers. Google Scholar was also searched for additional papers. The detailed strategy on PubMed was the following MeSH (Medical Subject Headings): (“Wearable Electronic Devices” [Mesh] OR wearable* [tiab] OR “Smart Band*” [tiab] OR “Smart Watch*” [tiab] OR wristband OR ((device* [ti] OR sensor* [tiab] OR sensing [tiab] OR biosensor* [tiab]) AND (wear* [tiab] OR worn [tiab]))) AND (“menstruation” [MeSH Terms] OR “Menstrual Cycle” [Mesh] OR “Fertility” [Mesh] OR menstruat* [tiab] OR Menstruation [tiab] OR menarche [tiab] OR menstrual period [tiab] OR menstru* [tiab] OR menses [tiab] OR catamenia [tiab] OR menarche [tiab] OR ovulation [tiab] OR ovulate [tiab] OR ovulatory [tiab] or “fertile window” [tiab] OR fertile [tiab] OR fertility [tiab] OR “LH surge” [tiab] OR “luteinizing hormone surge” [tiab] OR “luteal phase” [tiab]).

Gray literature was not included in this review in an attempt to only include peer-reviewed studies. This timeframe was chosen to reflect advances in smart sensors, artificial intelligence technologies, and their fertility tracking applications in medicine.

### Inclusion and Exclusion Criteria

All studies that evaluated an emerging wearable technology device (developed within the last 10 years) for fertility cycle tracking were included. Nonwearable devices, such as mobile health apps without a paired wearable device, personal digital assistants, or digital variations of thermometers that were nonwearable, were excluded. Two reviewers (LL and ME) screened the titles, abstracts, and full texts independently for potentially eligible studies. Reference lists of eligible studies were also hand-searched, but no additional studies were included on this basis.

### Study Selection and Data Extraction

Titles were screened for eligibility followed by abstracts. Each potential study for inclusion underwent full-text screening and was assessed for final eligibility and to extract study-specific data. Data were extracted and summarized in tabular format. For each of the included papers, we extracted information for the following: the year the paper was published, authors, authors’ country, study purpose, study design, the technology used, the bodily location of the technology placement, clinical measurement, and evaluation metrics. The outcome was evaluated based on the assessment measures (accuracy and statistical tests) used by each study to identify menstrual cycle stages. The number of participants across the studies was of interest when assessing the overall strength or reliability of each study. Two reviewers verified the extracted data independently.

## Results

### Study Selection

**Figure 1:**
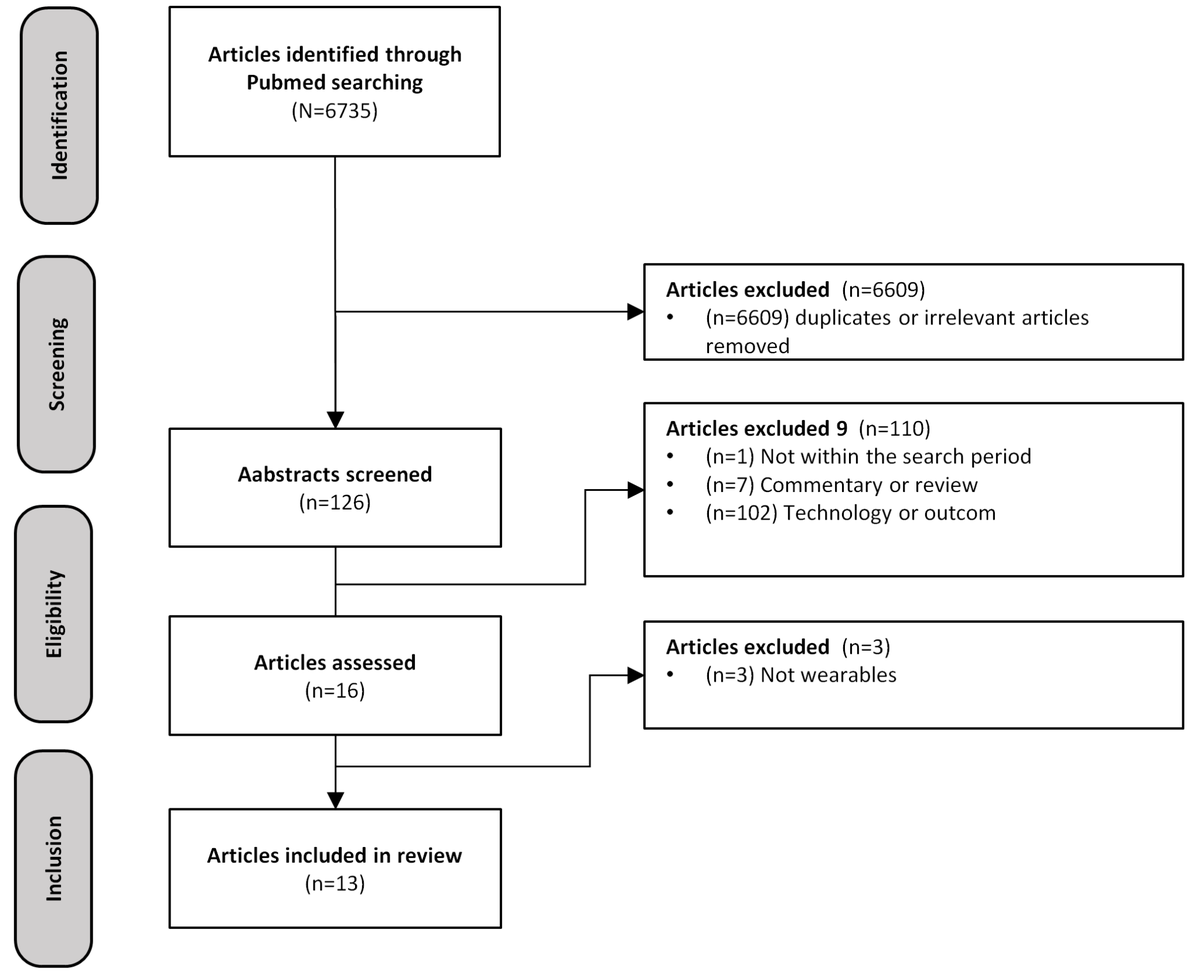
PRISMA (Preferred Reporting Items for Systematic Reviews and Meta-Analyses) flowchart.

A total of 13 studies met the inclusion criteria and were included in the review [7-13,24-28]. The flowchart illustrating the search process is summarized in Figure 1. Throughout the studies, changes in several physiological parameters were noted at different stages of the menstrual cycle. Notably, changes in HR, HRV, skin temperature, and respiratory rate were observed. Most of the studies evaluated wearable technology in the form of jewelry, such as the Oura Ring [7,9], or wrist-mounted sensors, such as the Ava bracelet [8,11-13].

A total of 2 studies used a wearable ear device to monitor skin temperature changes [25,26], and another 2 used an intravaginal biosensor (OvulaRing) [24,29]. Most used machine learning algorithms to predict stages of the menstrual cycle, including ovulation, based on physiological changes, such as temperature. LH levels were also objectively assessed via urine using Clearblue strips in most studies to confirm ovulation [7-13,22,24-28,30,31].

### Changes in Skin Temperature During the Cycle

Throughout the studies, changes in skin temperature during different phases of the menstrual cycle were recorded [7-9,12,13,25]. Most studies found that skin temperature increased during the early and late luteal phases [7-9,12]. Several studies have noted that skin temperature was higher during the luteal phase than during menstruation and ovulation [7,8]. For example, 1 study found that female individuals’ skin temperature is 0.33 °C higher during the luteal phase than during ovulation (fertile phase) (*P*<.001) [12]. Additionally, wrist skin temperature was found to be –0.72 °C lower during menstruation relative to the early luteal phase (*P*<.001) and –0.38°C lower than during the late luteal phase [12].

Additionally, studies have noted that temperature declined during the fertile phase, defined as 5 days leading up to and inclusive of ovulation, when compared with temperature during menstruation [8,13]. Goodale et al [8] found that skin temperature was 0.23 °C lower during the fertile window when compared with menstruation. The study by Zhu et al [13] found that body temperature increased after ovulation by 0.50 °C compared with basal body temperature increases of 0.20 °C, which was followed by a drop in temperature during menstruation of 0.33 °C using the skin temperature measure and 0.04 °C using basal body temperature. They also found that wrist-measured skin temperature was more sensitive and effective at measuring temperature changes during the cycle, with more pronounced temperature readings than basal body temperature, which was influenced by external factors, such as sleep, exercise, sexual intercourse, and caffeine intake [13].

### HR and HRV

In addition to body temperature, several studies have reported an increased HR during the luteal phase [8,11]. This was found when comparing HR during the luteal phase with both the menstruation and ovulation phases. For instance, 1 study found that female individuals in the midluteal phase of their cycle had a higher HR than during menstruation by 3.8 beats per minute (*P*<.001) [11]. Another study found that the nocturnal HR increased by 2.55 beats per minute during the late luteal phase relative to menstruation (*P*<.001), but the HRV ratio was reduced (–0.16) at nighttime (*P*=.004) [8].

Additionally, HR was found to increase during ovulation relative to menstruation [11]. For example, the study by Shilaih et al [11] found a higher HR of 2.1 beats per minute in ovulating female individuals when compared with menstruating female individuals (*P*<.001). The relationship was not as significant in female individuals with cycle irregularity [11]. In another study by Goodale et al [8], HRV was higher by 0.08 beats per minute (fertile) relative to menstruation. In addition to HR, 1 study evaluated respiratory rate and found that it increased during the late luteal phase by 0.18 breaths per minute relative to menstruation (*P*<.001) [8]. While the respiratory rate was reduced during the fertile period (follicular phase, ovulation, and early luteal phases) relative to menstruation, it was lower during menstruation relative to the late luteal phase [8]. This study also found that late-night meals and alcohol intake were all predictors of a rise in respiratory rate in female individuals [8].

### Validity

We conducted a validity assessment to evaluate the accuracy and reliability of measurements or data obtained by wearable technology for assessing fertility.

Alzueta et al [7] found that the measurements of both nightly HRs in tandem with body temperature are statistically significantly elevated (*P*=.001 for HR and *P*<.001 for body temperature) during the luteal phases (mid to late) when compared with other phases of the menstrual cycle particularly menses. This suggests that there is a valid and measurable difference in HR and body temperature across different phases of the menstrual cycle.

Shilaih et al [11] further reinforced the validity of physiological measurements by reporting a significant difference in pulse rate between different phases of the menstrual cycle, with rising levels during ovulation compared with menstruation and notably during the midluteal phase. Additionally, their findings, along with a similar study on body temperature differences (Shilaih et al [12]), emphasize the robustness and accuracy of these measurements in tracking and understanding physiological changes throughout the menstrual cycle. Maijala et al [9] also found a statistically significant correlation between skin and oral temperature (measured via a thermometer; *r*=0.563; *P*<.001) [9]. Conversely, another study comparing the ActiGraph with the Ava bracelet found that the Ava bracelet had limited predictive ability in assessing changes in HR in female individuals (*r*=–0.28; *P*<.001) [10].

The sensitivity for detecting fertility ranged from 62% [13] for a wrist-worn sensor to 92% for an ear-worn device [28] and an abdominal sensor worn at night [25]. The study by Maijala et al [9] found that the Oura Ring had a sensitivity of 83% for detecting ovulation and a sensitivity that ranged from 71.9% to 86.5% for detecting menstruation. Similarly, OvulaRing, a vaginal biosensor that measures internal body temperature changes in relation to fertility, has an accuracy of 88% when measured during the fertile period (3 days before ovulation, during ovulation, and 3 days following) [29]. An inner ear device with a machine learning algorithm that measured nighttime temperature changes had a sensitivity of 92.3% for tracking fertility relative to conventional methods [25]. The Ava bracelet, which measures HR and body temperature changes, had an accuracy of 90% in 1 study for detecting the fertile period, defined as 5 days before and during ovulation [8]. However, the study authors did not benchmark the device against the gold standard with regard to heart assessment [8]. Another study of the Ava bracelet found that it was more sensitive to predicting fertility than conventional methods, which involved basal body temperature checks, with a reported sensitivity of 62% versus 23% [13].

## Discussion

### Principal Findings

This review aimed to establish a better understanding of the emerging wearables that have been developed, evaluated, and validated for menstrual cycle tracking including premenstruation, menstruation, and the fertile period (ovulation). Although we found that most wearables were worn externally on the wrist or finger, other emerging developments have included internally worn devices inside the ear and vagina to measure core body temperature rather than skin temperature. Most studies found that their devices were able to accurately detect physiological changes in temperature and HR in relation to the menstrual cycle stage. Only 1 study evaluated a wearable in relation to respiratory rate.

Interestingly, 1 study found that skin temperature is more accurate as a proxy for menstrual cycle stage estimation [13], given that wearing it on the wrist is less sensitive to other external stimuli [13] and may be evaluated continuously when the user is sleeping. Evaluating changes while sleeping may also have practical benefits if the fertility monitoring wearable is comfortable and the user does not need to wear the device continuously during the day.

Although wearables for menstruation tracking are still being developed, and more studies are needed to build the evidence base, it appears that they are a promising method for tracking fertility in female individuals. Such devices could be used as part of an array of products that promote the reproductive health of female individuals. Thus, they could be widely used as a method of tracking the fertile period and helping female individuals make informed decisions about their reproductive health, including avoiding conception or increasing their chances of conception around their peak fertility dates.

In addition to using these devices to track the fertile period, insight into the timing of menstruation may also be beneficial for some female individuals, such as those who experience premenstrual dysphoric dysfunction—a condition that causes anxiety, anger, irritability, and pain [32] 1 week before menstruation [33]. Such a condition is relevant as it occurs during the luteal phase [34], which can be tracked with fertility wearable devices. Additionally, some countries have implemented days off work for female individuals when they menstruate, such as Japan [35].

Premenstrual dysphoric disorder is also associated with worsening depression and anxiety, although it is clinically distinct [36]. Knowing when female individuals with premenstrual dysphoric disorder experience symptoms based on cycle tracking may help empower them through heightened awareness and help them plan their schedules accordingly. Likewise, female individuals with dysmenorrhea (prevalence from 45% to 95%), which is painful cramping during menses, may experience disruption to their daily activities [37,38]. By precisely timing the date of their period, they may be able to plan their social and work schedules more efficiently and precisely.

One question of interest is whether wearables may be a better alternative to using calendars or digital apps. While studies have found that wearables are less accurate for female individuals who experience menstrual cycle irregularity relative to their regular counterparts [11,12], they nonetheless may provide an advantage over diary entry methods in this population. In other words, female individuals who have irregular cycles may, in fact, benefit from gaining insight into the predicted fertile dates based on their body temperature, HR, and respiratory rate, rather than relying on an electronic diary entry of past dates, which may not predict future ovulatory dates. In addition to this, recently, the Clue app was Food and Drug Administration approved and involves menstruation tracking through at-home ovulation test kits for detecting peaks in LH along with calendar tracking [39]. Thus, combined methods may perhaps be the best option, but research is needed to investigate this.

### Skin Temperature Versus Basal Body Temperature Versus Vaginal Temperature

By contrast, intravaginal temperature monitoring has been found to be a good proxy for measuring core body temperature, especially with menstrual cycle monitoring [40]. It is interesting to note that 1 study in our review found that skin temperature was more accurate than basal body temperature and was one of the few prospective comparative efficacy studies, but it was worn on the wrist at night [13]. It is important to note that there are other factors that need to be considered including the comfort of the female individual who will be using these devices. It may not be as comfortable for female individuals to assess their core basal body temperature using an intravaginal monitor when compared with less invasive ear temperature or skin wrist temperature assessments, for example. Thus, there is a trade-off between comfort and full detection accuracy, something that future wearable companies should keep in mind when designing their devices to maximize accuracy and minimize any potential physical or psychological discomfort for the user.

Additionally, the fertile period takes place 5 days prior to ovulation [16,19], but the OvulaRing can only detect 3 days prior to ovulation, which is a major limitation as sperm may still fertilize an egg due to their 5-day viability [29]. Likewise, the Oura Ring can detect the fertile window 3 days prior to ovulation and 2 days after [9]. Detecting fertility 3 days after ovulation [29] is not of much use as the egg remains viable for only 24 hours after it is released [16,19]. This means that female consumers should be aware of these limitations as pregnancy may still occur in the 2 days prior. In addition to this, even female individuals with regular cycles may experience changes in their cycle as a result of stress [40] or an infection [41], including COVID-19, and elevated body temperature from infection [41] may further misconstrue the findings.

Moreover, the study designs had limitations given several were mostly 1-armed pilot studies with small sample sizes. There were some studies that were prospective with larger sample sizes that followed many cycles [8] or compared 2 different fertility tracking methods [13], for example (refer to Table 1). However, there is a need for more studies that will follow the gold standard including comparative prospective efficacy studies and randomized controlled trials [42] that will be sufficiently powered with large enough sample sizes to compare current wearables with other methods for tracking fertility. Recently, the Food and Drug Administration cleared the Nature Cycles app for fertility monitoring in female individuals [43]. However, due to a lack of sufficient efficacy, a recent lawsuit resulted from unplanned pregnancy in female individuals who had been using it [43]. This highlights the need to undertake rigorous efficacy studies before these digital fertility tracking products are commercially marketed directly to consumers.

Based on this study, intravaginal temperature monitoring is considered a good proxy for measuring core body temperature, particularly during menstrual cycle monitoring. However, 1 study found skin temperature to be more accurate than basal body temperature, albeit with limitations in wrist-worn devices. Comfort is also an important factor for users, as intravaginal monitoring may be less comfortable compared with less invasive methods like ear and skin temperature assessments. Our study also highlights the detection windows of different wearables, such as the OvulaRing and Oura Ring, and the need for more comprehensive comparative studies. Ensuring rigorous efficacy studies before marketing fertility tracking products to consumers is essential to avoid unforeseen consequences.

**Table 1:** Summary of the included studies.

Year	Study	Sample size, N	Technology	Measures	Results
2022	Alzueta et al [7]	26	Oura Ring	HR^a^, HRV^b^, and distal temperature	HR was higher during the midluteal phase vs menstruation (*P*=.001) Higher HR during late luteal (*P*=.001) vs menses Ovulation linked with lower HR vs luteal phases including midluteal (*P*=.02) and late luteal phases (*P*=.01) Significant rise in body temperature during mid- and late luteal phases compared with menstruation and ovulation (*P*<.001) Marginally lower body temperature during ovulation compared with menstruation (*P*=.05)
2019	Maijala et al [9]	22	Oura Ring	Nocturnal finger skin temperature	Higher skin temperature during luteal phase vs follicular (0.30 °C, SD 0.12), and compared to oral temperature during this phase of increase (0.23 °C, SD 0.09; *P*<.001) Sensitivity for ovulation detection using the Oura ring=83.3% (–3 to +2 days) Sensitivity for menstruation detection=71.9%-86.5% (SD 2-4 days) Skin temperature correlation with oral temperature (thermometer); r=0.563 (*P*<.001) Differentiating cycle stages; *r*=0.589 (*P*=.004)
2022	Nulty et al [10]	33	Ava bracelet	Skin temperature, resting pulse rate, HRV ratio, skin perfusion, breathing rate, and movement	Acceptability for ovulation tracking is high, 19.3/25 (77%), with poor predictive ability HRV compared with HR chest strap for direct heart monitoring + ActiGraph Mean percentage error HRV=11.4% Correlation against gold standard, –r=–0.28 (*P*<.001)
2018	Regidor et al [29]	158	OvulaRing	Core body temperature	Accuracy is 88% for detecting ovulation (SD of 3 days around ovulation)
2017	Shilaih et al [11]	91	Wrist-worn photoplethysmography sensor	HR or pulse rate	Rise in pulse rate during ovulatory phase relative to menstruation (median): +2.1 beats/min (*P*<.001) Midluteal phase: pulse rate highest vs ovulation (fertile period): + 1.8 beats/min (*P*<.01) and relative to menses :3.8 beats/ min (*P*<.001)
2018	Shilaih et al [12]	136	Wrist-worn photoplethysmography sensor: Ava bracelet	Body temperature (wrist skin)	Increased temperature on the wrist during luteal phase (+0.33 °C relative to ovulation [fertile window]); *P*<.001 Lower wrist temperature during menstruation than during luteal phases including late luteal phase (–0.38 °C; *P*<.001) Change in temperature during the fertile period=86%
2019	Goodale et al [8]	193	Ava wrist bracelet	HRV, HR, respiratory rate, and wrist skin temperature	Wrist temperature significantly lower during the fertile phase relative to menstruation (b=–0.25, SD 0.03; *P*<.001) HR lower during fertile phase compared with menstruation (b=–0.03, SD 0.26; not significant) HRV significantly higher during fertile stage (b=0.08, SD 0.03; *P*<.05) relative to the comparison of menstruation Lower respiration rate during fertile period vs menses (b=–0.48, SD 0.04; *P*<.01) HR is higher in luteal and midluteal relative to menstruation (*P*<.01) Late luteal phase relative to menses increases in respiratory rate by 0.18 breaths per min (*P*<.001) Accuracy for detecting fertility=90%
2019	Luo et al [25]	34	Ear device	Temperature	Ovulation detection Sensitivity =92.3% Increased predictive power 23%-31.5% Compared to conventional methods of fertility tracking (kit)
2021	Zhu et al [13]	63	Ava fertility bracelet	Wrist skin temperature and basal body temperature	Wrist skin temperature decreases during menstruation (0.33 °C vs 0.04 °C for basal body temperature) Sensitivity higher for the wrist temperature measure vs the standard basal body temp (oral thermometer) measure (0.62 compared to 0.23; *P*<.001) False positive rate greater for wrist skin temperature by 5.2% compared with basal body temperature
2022	Hurst et al [24]	80	Skin worn sensor + vaginal biosensor algorithm, OvuSense, OvuCore, OvuFirst	Body temperature	Accuracy for ovulation prediction=66% (SD 1 day) 90% accuracy for the fertile window (ovulation SD 3 days)
2020	Prasannan and Sarath [26]	30	Internet of things device with a flex sensor worn on the abdomen	Body temperature	ovulation prediction Accuracy=92%
2022	Yu et al [27]	114	Huawei band + ear thermometer	Body temperature and HR	Fertility window Accuracy=87.4% Menstruation cycle Accuracy=89.6%
2009	Chen et al [28]	30	Abdominal sensor worn during sleep	Body temperature	Menstrual cycle stage sensitivity=91.8% PPV^c^=96.6%

^a^HR: heart rate.

^b^HRV: heart rate variability.

^c^PPV: positive predictive value.

### Study Quality and Design Assessment

There are several limitations with wearables for fertility tracking. First, some of the studies on temperature validated their findings and reported relatively good accuracy, but not all studies did, which limits our overall assessment of validity. Second, many used skin temperatures to assess changes in the menstrual cycle. Research indicates that skin temperature may not be as accurate as basal body temperature because it is influenced by the external environment and resultant physiological changes such as perspiration from heat or physical activity [40]. Research has found that intraoral and ear-based temperature methods are less accurate for predicting menstrual cycle phases [44]. Several studies have explored the potential of wearable devices for fertility and menstrual cycle tracking. Alzueta et al [7] provided valuable insights with their study involving 26 participants and the use of the Oura Ring to measure HR, HRV, and distal temperature. Similarly, Maijala et al [9] reported encouraging sensitivity and positive predictive value for nocturnal skin temperature with 22 participants using the Oura Ring, although the small sample size may limit generalizability. Nulty et al [10] raised questions about the Ava bracelet’s HRV as a predictor for fertility tracking in their study with 35 participants. However, larger-scale studies and accounting for potential confounders could enhance the study quality. On a more promising note, Regidor et al [29] conducted a study with 158 participants, evaluating the accuracy of OvulaRing in measuring core body temperature, reporting promising results that warrant further consideration and addressing potential biases. Shilaih et al [11,12] conducted 2 studies with 91 and 136 participants, respectively, using wrist-worn photoplethysmography sensors and the Ava bracelet to measure changes in HR or pulse rate and body temperature (skin) throughout the cycle. The observed significant differences in pulse rate and body temperature between menstrual phases are intriguing and should be further investigated by controlling for confounders. Goodale et al [8] conducted a study with 193 participants, using the Ava wrist bracelet to measure HRV, HR, respiratory rate, and wrist skin temperature. The study provided valuable insights into the changes observed across the menstrual cycle, and future research should address potential biases related to participants’ daily activities and lifestyle factors affecting the measured parameters. Luo et al [25] reported high sensitivity in detecting ovulation using an ear device for temperature measurement involving a total of 34 participants. Zhu et al [13] explored the effectiveness of the Ava fertility bracelet in detecting ovulation based on wrist skin temperature and basal body temperature with 63 participants. The reported higher sensitivity for wrist skin temperature is intriguing and merits further investigation to address potential biases related to device placement and calibration. Similarly, Hurst et al [24] evaluated a combination of skin-worn sensors, vaginal biosensors, and algorithms for predicting varying menstrual cycle stages with a total of 80 participants, demonstrating promising accuracy for predicting fertile periods. However, potential biases related to algorithm development and data processing should be addressed to ensure reliable results. Prasannan and Sarath [26] applied an internet of things device with a flex sensor worn on the abdomen to evaluate body temperature with a total of 30 participants. While notable accuracy for ovulation prediction was observed, future studies should consider potential biases related to sensor placement and participant compliance. Finally, Yu et al [27] conducted a study with 114 participants, using the Huawei band with an ear thermometer to measure body temperature along with HR. The reported accuracy, sensitivity, and specificity for fertility and menstruation are encouraging, but future research should address potential biases related to device calibration and participant adherence to enhance the reliability of the findings.

Wearable fertility tracking studies offer valuable insights, but their study design, potential biases, and limitations should be carefully considered. Future research with larger sample sizes, more rigorous designs, and controlled conditions will contribute to a better understanding of the reliability and effectiveness of wearable technologies in fertility tracking.

### Future Research

Furthermore, more qualitative consumer-focused research is needed to learn about the experiences of female individuals who use these devices to improve the technology, address any barriers, and promote the facilitators of use. Little is known about whether female individuals find these devices comfortable to wear, if they have any specific design preferences, or whether they are interested in using these emerging technological devices for long-term fertility and menstrual cycle monitoring. Privacy [45] and ethical issues around the digital health monitoring of fertility have been especially relevant during the time of Roe v. Wade in the United States, where many female individuals no longer feel safe sharing their private information concerning their menstrual cycles [46]. Thus, it is especially prudent to ensure that the privacy of the data associated with wearable technology is secured. It is also important to determine whether this form of fertility monitoring feels acceptable and useful for female individuals in the United States in the current climate.

Finally, future research should involve more studies to expand the literature base that will involve quality trials comparing the wearables with other methods for fertility detection with larger sample sizes. There is a clear need for well-designed efficacy studies for both pregnancy prevention and time to pregnancy in the future.

A limitation of our review is that we focused on wearables that have been scientifically assessed in our MEDLINE or PubMed search. There is a need for broader research into all commercially available devices in the future for female individuals to be well informed about other products that they may be using on the market, but there is also a need for more evidence-based efficacy studies to support them.

### Conclusions

In summary, we reviewed wearable technology devices for fertility cycle monitoring. Although there are not many studies on this topic, the emerging research suggests that these devices hold promise as an accurate method of detecting fertility cycle stages, including the fertile window before and during ovulation, menstruation, and differentiation of the luteal phases (early to late). Most studies assessed physiological changes, such as temperature, and a combination of changes that included HR and HRV. Respiratory rate was also used to predict fertility cycle stages, but more research is needed to validate this method. Overall, the studies found that these emerging wearables are promising for menstrual cycle monitoring. Wearables for fertility cycle monitoring appear to be a valuable adjunct to the current arsenal of fertility tracking and reproductive health available to female individuals. However, more research is needed on the consumer acceptability of these devices and how to protect female individuals’ privacy best when wearing digital devices that collect continuous data on their reproductive health. There is also a need for more comparative prospective efficacy studies to validate these devices for their efficacy.

## Appendix

Multimedia Appendix 1 PRISMA (Preferred Reporting Items for Systematic Reviews and Meta-Analyses) checklist.

## Abbreviations

HR: heart rate
HRV: heart rate variability
LH: luteinizing hormones
MeSH: Medical Subject Headings
PRISMA: Preferred Reporting Items for Systematic Reviews and Meta-Analyses

## Data Availability

All data generated or analyzed during this study are included in this published paper.
